# Supplementary material for: A click-based electrocorticographic brain-computer interface enables long-term high-performance switch scan spelling
Source: Commun Med (Lond). 2024 Oct 22;4:207. doi: 10.1038/s43856-024-00635-3 (PMC11494178; doi:10.1038/s43856-024-00635-3)
Supplement: Supplementary file 2 — Supplementary Material [file 43856_2024_635_MOESM2_ESM.pdf]

A click-based electrocorticographic brain-computer interface enables long-term high-performance switch scan spelling

Supplementary Materials

Daniel N. Candrea[1✉], Samyak Shah[2], Shiyu Luo[1], Miguel Angrick[2], Qinwan Rabbani[3], Christopher Coogan[2], Griffin W. Milsap[4], Kevin C. Nathan[2], Brock A. Wester[4], William S. Anderson[5], Kathryn R. Rosenblatt[6], Alpa Uchil[2], Lora Clawson[2], Nicholas J. Maragakis[2], Mariska J. Vansteensel[7], Francesco V. Tenore[4], Nicolas F. Ramsey[7], Matthew S. Fifer[4], Nathan E. Crone[2]

✉Daniel N. Candrea: dcandre3@jh.edu

1 Department of Biomedical Engineering, Johns Hopkins University School of Medicine, Baltimore, MD

2 Department of Neurology, Johns Hopkins University School of Medicine, Baltimore, MD

3 Department of Electrical and Computer Engineering, Johns Hopkins University, Baltimore, MD

4 Research and Exploratory Development Department, Johns Hopkins University Applied Physics Laboratory, Laurel, MD

5 Department of Neurosurgery, Johns Hopkins University School of Medicine, Baltimore, MD

6 Department of Anesthesiology and Critical Care Medicine, Johns Hopkins University School of Medicine, Baltimore, MD

7 Department of Neurology and Neurosurgery, UMC Utrecht Brain Center, Utrecht, The Netherlands

Table of Contents

Supplementary Notes

Supplementary Note 1 | Recording viability of the study device. Page 4

Supplementary Note 2 | BCI functionality of the study device. Page 4

Supplementary Note 3 | Other assistive devices used by the participant. Page 5

Supplementary Note 4 | ALS functional rating scale (ALSFRS-R). Page 5

Supplementary Note 5| Retraining a new click detector after performance drop. Page 9

Supplementary Note 6 | Selecting the optimal voting threshold. Page 9

Supplementary Note 7 | Electrode contributions to attempted grasp during transient performance drop. Page 10

Supplementary Note 8 | Decreasing preparatory periods may improve spelling rate. Page 10

Supplementary Methods

Supplementary Method 1 | Clinical trial inclusion and exclusion criteria. Page 11

Supplementary Figures

Supplementary Fig. 1 | Training paradigm. Page 13

Supplementary Fig. 2 | Cue-aligned spectrograms of upper-limb channels. Page 14

Supplementary Fig. 3 | Alignment of power trial rasters. Page 15

Supplementary Fig. 4 | Correlation increase of HG power in re-aligned signals. Page 16

Supplementary Fig. 5 | Correlation analysis of channel 112 using re-aligned trials. Page 17

Supplementary Fig. 6 | Labelling trial-averaged re-aligned HG power. Page 18

Supplementary Fig. 7 | Classification model architecture. Page 19

Supplementary Fig. 8 | User interface of communication board. Page 20

Supplementary Fig. 9 | User interface of switch scanning speller application. Page 21

Supplementary Fig. 10 |Offline confusion matrices of spelling blocks. Page 22

Supplementary Fig. 11 | Click detector performance as a function of training data. Page 23

Supplementary Fig. 12 | Click detector performance using updated models. Page 24

Supplementary Fig. 13 | Timelines of click use after training. Page 25

Supplementary Fig. 14 | Performance decline roughly 4 months post-training. Page 26

Supplementary Fig. 15 | Decrease in movement-aligned high gamma (HG) power. Page 27

Supplementary Fig. 16 | Increase in magnitude of movement-aligned low frequency power. Page 28

Supplementary Fig. 17 | Long-term use of a fixed retrained click detector. Page 29

Supplementary Fig. 18 | Performance evaluation across all voting thresholds. Page 30

Supplementary Fig. 19 | Switch scan spelling performance with a retrained fixed click detector. Page 31

Supplementary Fig. 20 | Normalized saliency values. Page 32

Supplementary Fig. 21 | Normalized saliency values during period with performance drop. Page 33

Supplementary Tables

Supplementary Table 1 | Experimental parameters for training data collection. Page 34

References

Page 35

Supplementary Notes

Supplementary Note 1. Recording viability of the study device.

The implant recording viability was assessed by visual inspection of the channel raw voltage signals as well as by measuring electrode impedances. Visual inspection of the voltage signals occurred at the beginning of each session, while electrode impedances were measured once per week.

Voltage signals were visually inspected for the presence of noise, signal amplitudes exceeding the digital bit-range of the recording hardware, signals with unusually low amplitude, or presence of movement artifact. Throughout the study, there were no fewer than 124 viable channels. Suboptimal signal quality was observed in four channels (channels 19, 38, 48 and 52) all of which were located on the electrode grid covering cortical speech areas (see Fig. 1b for anatomical locations). However, only channel 38 was consistently marked for suboptimal signal quality throughout the duration of the trial.

It is important to note that the decision to exclude channel 38 from model training was made only after the initial click detection algorithm was deployed for online use. Meanwhile channels 19, 48, and 52 were never marked for poor signal quality during data collection for initial model training but were marked as such later in the trial. The click detection algorithm trained without features from these channels (Supplementary Note 5) performed comparably to the model trained with these channel features included (see *Click detector retraining due to transient performance drop*). Indeed, features from these four channels provided minimal contributions to classification (Supplementary Fig. 20) and by themselves produced a mean classification accuracy of 55.2% (chance 50%) using repeated 10-fold cross validation (see *Channel contributions and offline classification comparisons*).

Electrode impedances were measured using the Impedance Tester tool on Central Suite (Blackrock Neurotech Corp.). Particularly, electrodes whose impedance values exceeded a 15 kΩ threshold by an order of magnitude were compared to channels with suboptimal signal quality and would be additionally excluded from analysis if they were not already marked by visual inspection. To date, only channel 38 has presented with an impedance consistently roughly two orders of magnitude higher than the 15 kΩ threshold. On one session channel 121 also presented with a similarly high impedance.

Supplementary Note 2. BCI functionality of the study device.

The goal of this aspect of the study was to demonstrate control of external devices through speech and/or motor strategies, as well as to assess performance of each strategy. Because of the exploratory nature of the study and limited number of participants, the specific strategies, tasks, performance metrics, and longitudinal assessments were not predefined. Rather, we anticipated that these methods would evolve during the trial and would be customized to each participant. Likewise, we did not formalize a statistical analysis plan in the protocol. In keeping with this protocol, the present work demonstrates a proof-of-concept spelling system controlled by attempted grasping movements and an ECoG-based BCI in a single participant. Our approach to reporting these results is similar to that of numerous published single-subject studies of implantable BCI functionality[1–6].

Supplementary Note 3: Other assistive devices used by the participant.

During the study described in this writing, it was not necessary for the participant to rely on his BCI, and he mostly did not need to rely on other assistive communication devices at home. Due to his slowly progressing condition, he could still verbally communicate with the experimental team and his family, albeit laboriously and with limited intelligibility. Further, the participant retained eye movements and residual movements in both his upper and lower extremities but could not perform activities of daily living without assistance.

When the participant did use an assistive communication device at home, he used primarily his Tobii Dynavox (Danderyd, Sweden) or cell phone. The participant used the Tobii Dynavox's virtual keyboard (QWERTY layout) for typing and used eye-tracking to select letters and predictive words. Additionally, the participant would also type slowly on his cell phone using primarily his index finger, and he used the text-to-speech feature to synthesize his text to audio. He used these methods infrequently.

During the study, the participant reported that it was still easier to control the Tobii Dynavox's spelling application via eye control than it was to control the BCI-based spelling application. His eye movements were not yet significantly affected by ALS. Nevertheless, eye fatigue would require him to pause the application for a few minutes every 10-15 minutes. Also, jitter in the eye cursor was more pronounced when he was fatigued. Finally, changes in the ambient lighting sometimes required him to adjust his eye movements to correct for unintended cursor deviations.

Supplementary Note 4. ALS Functional Rating Scale (ALSFRS-R)

The clinical care team at Johns Hopkins Hospital obtained the assessment for the ALSFRS-R measure one day before we started collection of training data. In total, the study participant in the clinical trial scored 26 out of 48 points. The scores for each category of the ALSFRS-R are shown below in bold.

1. SPEECH

4. Normal speech processes
3. Detectable speech disturbances
2. Intelligible with repeating
1. Speech combined w/ nonvocal communication
0. Loss of useful speech

2. SALIVATION

4. Normal
3. Slight but definite excess of saliva in mouth; may have nighttime drooling
2. Moderately excessive salvia; may have minimal drooling
1. Marked excess of saliva with some drooling
0. Marked drooling; requires constant tissue or handkerchief

3. SWALLOWING

4. Normal eating habits

3. Early eating problems – occasional choking

2. Dietary consistency changes

1. Needs supplemental tube feedings

0. NPO (exclusively parenteral or enteral feeding)

4. HANDWRITING

4. Normal

3. Slow or sloppy; all words are legible

2. Not all words are legible

1. Able to grip pen but unable to write

0. Unable to grip pen

5a. CUTTING FOOD AND HANDLING UTENSILS

(Patients *without* gastrostomy) **NA**

4. Normal

3. Somewhat slow and clumsy; no help needed

2. Can cut most foods (clumsy and slow); some help needed

1. Foods must be cut by someone, but can still feed slowly

0. Needs to be fed

5b. CUTTING FOOD AND HANDLING UTENSILS

(Patients *with* gastrostomy)

4. Normal

3. Clumsy, but able to perform all manipulations independently

2. Some help needed with closures and fasteners

1. Provides minimal assistance to caregiver

0. Unable to perform any aspect of task

6. DRESSING AND HYGIENE

4. Normal

3. Independent self-care with effort or decreased efficiency

2. Intermittent assistance or substitute methods

1. Needs attendant for self-care

0. Total dependence

7. TURNING IN BED & ADJUSTING BEDCLOTHES

4. Normal

3. Somewhat slow and clumsy, but no help needed

2. Can turn alone or adjust sheets, with great difficulty

1. Can initiate, but not turn or adjust sheets alone

0. Helpless

8. WALKING

4. Normal

3. Early ambulation difficulties

2. Walks with assistance

1. Nonambulatory functional movement only

0. No purposeful leg movement

9. CLIMBING STAIRS

4. Normal

3. Slow

2. Mild unsteadiness or fatigue

1. Needs assistance

0. Cannot do

10. DYSPNEA (shortness of breath)

4. None

3. Occurs when walking

2. Occurs with one or more of the following: eating, bathing, dressing

1. Occurs at rest, either sitting or lying

0. Significant difficulty, considering using mechanical support

11. ORTHOPNEA (trouble breathing when lying flat)

4. None

3. Some difficulty sleeping at night due to shortness of breath; does not routinely use more than 2 pillows

2. Needs extra pillows in order to sleep (more than 2)

1. Can only sleep sitting up

0. Unable to sleep

12. RESPIRATORY INSUFFICIENCY

4. None

3. Intermittent use of BiPAP/NIV

2. Continuous use of BiPAP/NIV during the night

1. Continuous use of BiPAP/NIV during the night and day

0. Mechanical ventilation (intubation or tracheostomy)

Total: 26/48

Supplementary Note 5. Retraining a new click detector after performance drop.

We collected three blocks of training data using the Go task as described in *Training task*. Each block consisted of 60 trials during which the participant attempted to make a grasping motion, and each trial was followed by an interstimulus interval (ISI) during which the participant remained still and fixated his gaze on a crosshair in the center of the monitor (Supplementary Fig. 1). The ISI length was randomly chosen between 3.5-4.5 s. Each block started with a 30 s rest period during which the participant was instructed to remain still with his gaze fixated on a "Rest" stimulus. In total, 180 trials were collected for a total of almost 15 min of data. All three blocks were collected on the same day (Day 309 post-surgical implantation, Supplementary Fig. 17a). Neural signals were recorded by the Neuroport system with a sampling rate of 2 kHz. We excluded four channels (channels 19, 38, 48, and 52) due to poor raw voltage signal quality determined by visual inspection. High gamma (HG) features were computed and re-aligned to account for inter-trial variability as described in *Feature extraction and label assignment*. We assigned grasp labels to ECoG feature vectors falling between 0.4 s and 1.2 s post-cue for each trial and rest labels to all other feature vectors. We used the model architecture and techniques described in *Model architecture and training* to train our model. During spelling sessions, video of the participant's right hand was collected at 60 frames per second (FPS) during all sessions with the retrained algorithm.

Supplementary Note 6. Selecting the optimal voting threshold.

We defined the optimal voting threshold as the one that produced the highest F_1-score:

$$F_1 = \frac{2N_{TP}}{2N_{TP} + N_{FP} + N_{FN}}$$

where N_{TP} and N_{FP} are the number of true and false positive clicks, respectively, and N_{FN} is the number of missed clicks. N_{FN} can be rewritten as $N_{attempted\ grasps} - N_{TP}$, and so the F_1-score can be rewritten as:

$$F_1 = \frac{2N_{TP}}{N_{TP} + N_{FP} + N_{attempted\ grasps}}$$

The F_1-scores for all voting thresholds are calculated using the total numbers of attempted grasps, true and false positives across all six sessions with the retrained click detector:

Voting threshold	2 votes	3 votes	4 votes	5 votes	6 votes	7 votes
$N_{attempted\ grasps}$	597	582	476	421	423	461
N_{TP}	563	519	435	382	399	382
N_{FP}	57	23	20	13	14	0
F_1-score	0.925	0.923	0.934	0.936	**0.955**	0.906

The 6-vote threshold produced the highest F_1-score of 0.955.

Supplementary Note 7: Electrode contributions to grasp classification during observed drop in performance.

We computed the importance of HG features from each channel when generating grasp during the time period when we observed a drop in click detector performance. We used largely the same procedure to label each sample as we describe in *Feature extraction and label assignment* with only two differences. First, we determined the onset and offset time of the re-aligned trial averaged HG power traces relative to the start of attempted movement rather than to a Go cue, which was not present during the spelling blocks. Second, since two attempted grasps could have occurred within a very short duration of each other (e.g., clicking into a row followed by clicking into the first column), we excluded from training all attempted grasps which occurred less than 3 s (the minimum jittered ISI, Supplementary Table 1) after a preceding attempted grasp. We then computed the integrated gradients (as described in *Channel contributions and offline classification comparisons*) from the original fixed click detector with respect to the input features from each sample labeled as grasp. This generated an attribution map for each sample[7]. The final saliency map was computed by averaging the attribution maps across all samples and normalizing the resulting mean values between 0 and 1.

Supplementary Note 8: Decreasing preparatory periods may improve spelling rate.

We included pre-selection rows and columns to allow the participant a brief preparation period before clicking into the first row or first column, respectively (Supplementary Fig. 9). However, the total time the switch scanner spent in these preparatory regions was 4 s per scanning cycle, during which the application could not receive clicks. For a future study participant, we may slowly start shortening these preparation periods and possibly eliminate them completely as the participant becomes more comfortable with timing his or her attempted grasps to highlighted rows or columns. Considering that we designed the application such that the language model's most probable words and letters appear in the top two "clickable" rows, reducing the total scan time by 4 sec per desired button could significantly increase the spelling rate. For example, assuming a TPF of 11 per min where half of the true positives were row clicks (succeeding preparatory periods of 3 s) and half were column clicks (succeeding preparatory periods of 1 s), the total time of the scanner spent in preparatory periods would be 22 s $\left(5.5\ \mathrm{TP} \times \frac{3\ \mathrm{s}}{\mathrm{TP}} + 5.5\ \mathrm{TP} \times \frac{1\ \mathrm{s}}{\mathrm{TP}}\right)$. Reducing these preparatory times to 0 s would have thus resulted in a TPF of 17.4 per min $\left(\frac{11\ \mathrm{TP}}{60\ \mathrm{s} - 22\ \mathrm{s}} \times \frac{60\ \mathrm{s}}{\mathrm{min}}\right)$. It could similarly be beneficial to rearrange the keyboard such that the most used letters take the fewest scans to reach (i.e., placement in the top left corner of the keyboard). Indeed, given a latency of 0.68 s from attempted grasp onset to click with the lock-out period of 1 s (1.68 s attributed to each click) a theoretical maximum TPF of 35 clicks per min could be approached with the appropriate user interface.

Supplementary Methods

Method 1. Clinical trial inclusion and exclusion criteria

Inclusion Criteria

Participants must meet all inclusion criteria, verified by medical evaluation, psychological evaluation, and review of medical history. Inclusion criteria include:

- Complete or incomplete tetraplegia (quadriplegia), tetraparesis (quadriparesis), severe ataxia, or moderate to severe motor impairments in both upper limbs, based on neurological exam. In addition, these motor impairments may be combined with severe motor-related speech impairment (dysarthria or anarthria), as in Locked In Syndrome (LIS) and amyotrophic lateral sclerosis (ALS), including the bulbar variant of ALS.
- Clinical diagnosis established for the etiology of motor impairments, including brainstem stroke[1], spinal cord injury, or progressive and irreversible neuromuscular disease, including amyotrophic lateral sclerosis (ALS).
- Persistence of motor impairments at least one year prior to enrollment if due to stroke or spinal cord injury
- 22-70 years
- Meeting surgical safety criteria, including surgical clearance by the participant’s primary healthcare provider, study physicians, and any necessary consultants
- Ability to communicate reliably, such as through eye movement
- Willingness and ability to provide informed consent
- Screened by rehabilitation psychologist with a result showing that the participant has a stable psychosocial support system with caregiver capable of monitoring participant throughout the study
- Ability and willingness to travel up to 100 miles to study location up to three days per week for the duration of the study
- Ability to understand and comply with study session instructions
- Participant consents to the study and still wishes to participate at the time of the study

Exclusion Criteria

All interested participants will be reviewed for the presence of exclusion criteria by medical evaluation, review of medical history, self (or assistant) report and psychiatric evaluation. Cognitive and psychological assessments will be comprised of established clinical tests such as the Mini-mental State Examination, Rey Auditory Verbal Learning Test, and Symptom Checklist-90-Revised Test. Presence of any of the following criteria will exclude participants from eligibility to participate. In addition, the medical team has the right to withdraw the participant at any time if any of the exclusion criteria emerge, and participants can withdraw at any time for any reason (see above). Exclusion criteria include:

• Performance on formal neuropsychological testing that indicates a significant psychiatric disorder or cognitive impairments that would interfere with obtaining informed consent or fully participating in study activities.

• Suicide attempt or persistent suicidal ideation within the past 12 months.

• Implanted devices that are incompatible with MRI, which may include pacemakers, cardiac defibrillators, spinal cord or vagal nerve stimulators, deep brain stimulators, and cochlear implants.

• History of substance abuse, narcotic dependence, or alcohol dependence in past 24 months

• Medical conditions contraindicating surgery of a chronically implanted device (e.g. osteomyelitis, diabetes, hepatitis, any autoimmune disease/disorder, epilepsy, skin disorders causing excessive skin sloughing or poor wound healing, blood or cardiac disorder requiring chronic anti-coagulation)

• Other chronic, unstable medical conditions that could interfere with subject participation.

• Presence of pre-surgical findings in anatomical, functional, and/or vascular neuroimaging that makes achieving implant locations within desired risk levels too challenging (to be decided by neurological and neurosurgical team)

• Prior cranioplasty

• Inability to undergo MRI or anticipated need for an MRI during the study period

• Participants with active infections or unexplained fever

• Participants with other morbid conditions making the implantation of the recording elements unsafe; not limited to: significant pulmonary, cardiovascular, metabolic, or renal impairments making the surgical procedure unsafe

• Pregnancy (confirmation through blood test)

• Nursing an infant, planning to become pregnant, or not using adequate birth control

• Corrected vision poorer than 20/100

• HIV or AIDS infection

• Existing scalp lesions or skin breakdown

• Chronic oral or intravenous use of steroids or immunosuppressive therapy

• Active cancer within the past year or requires chemotherapy

• Uncontrolled autonomic dysreflexia within the past 3 months

• Hydrocephalus with or without an implanted ventricular shunt

• Participants in whom it is medically contraindicated to stop anti-coagulant medications during surgery

Supplementary Figures

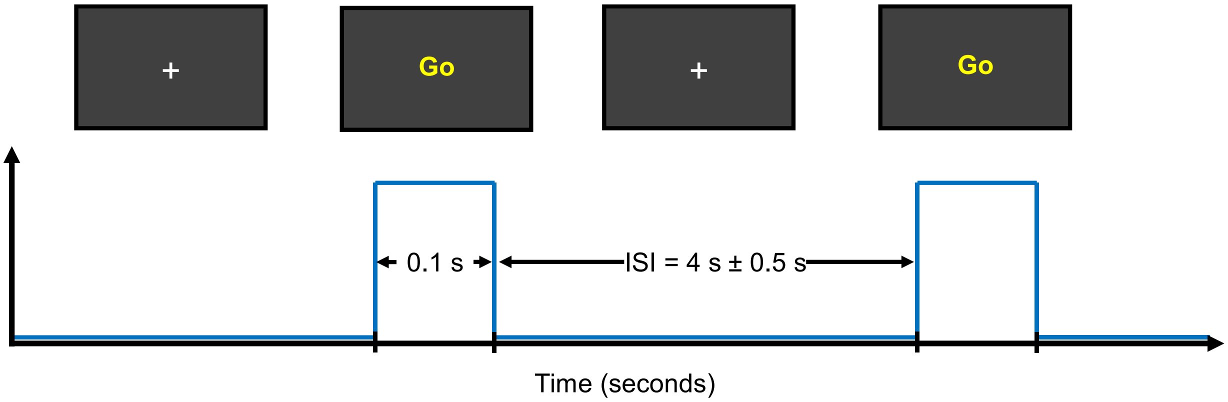

Supplementary Fig. 1 | Training paradigm. As the participant was seated in a chair with his forearms on the armrests, he was instructed to attempt a brisk grasp with his right hand immediately after the visual stimulus "Go" appeared on the monitor. One trial consisted of one 100 ms Go stimulus (t_{Go} = 0.1 s, not to scale for the purpose of clear depiction) followed by an interstimulus interval (ISI) during which a white crosshair appeared in the center of the monitor for the duration of the ISI. The length of each ISI was randomly chosen between a lower and upper bound (t_{ISI} = 3.5 - 4.5 s (as depicted) or 3 - 6 s, see Supplementary Table 1) to reduce anticipatory behavior.

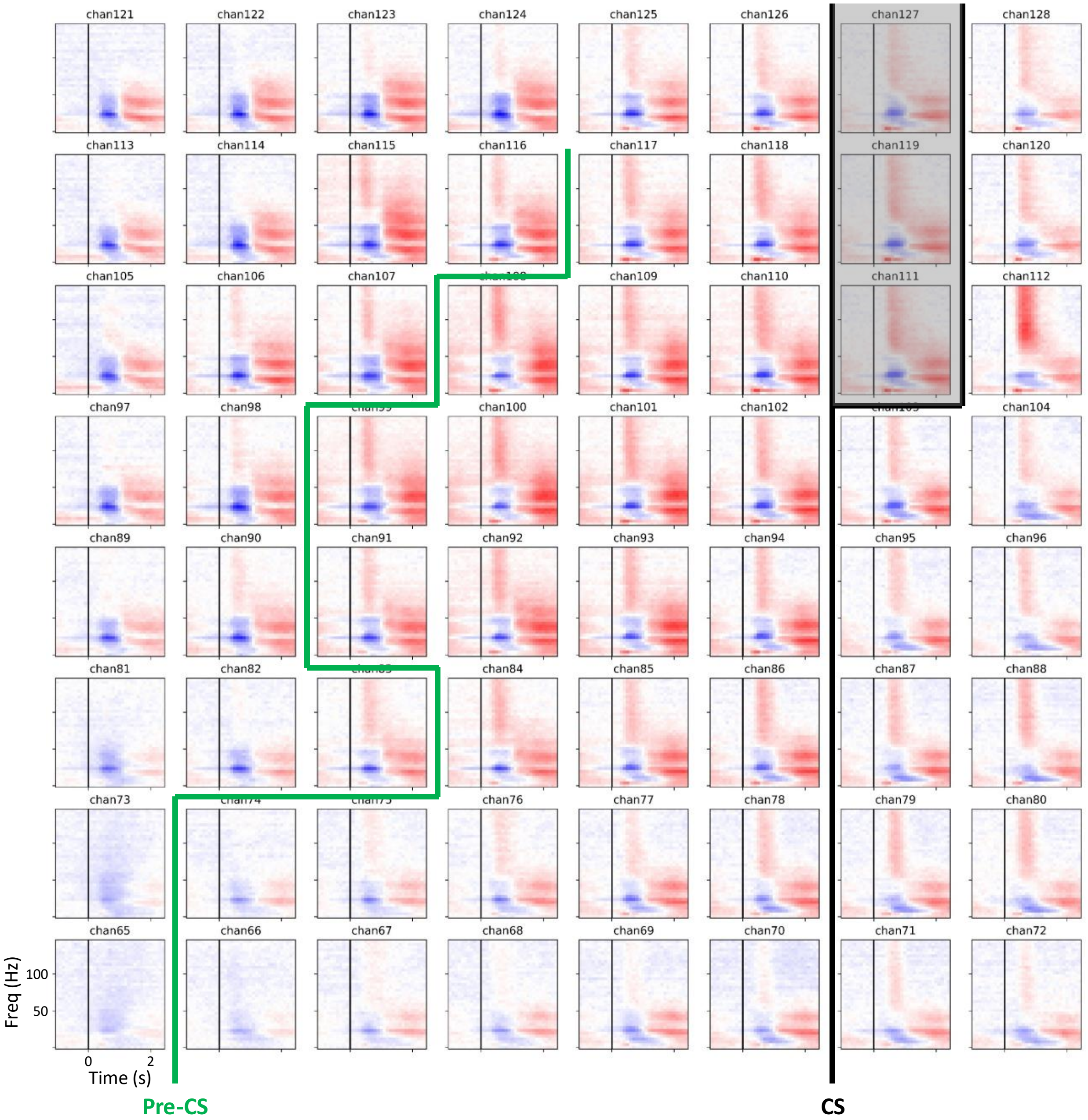

Supplementary Fig. 2 | Cue-aligned spectrograms of upper-limb channels. Across trials from all training blocks used for the original click detector, the cue-aligned trial-averaged spectrogram for all channels in the upper-limb grid is shown (-1 to 2.5 s post-cue). The vertical black lines for each channel represent the onset of the visual cue. Spectral power at each frequency is standardized to the statistics of the 1 min calibration period. The slightly increased broadband high frequency activity observed in some channels' pre-cue interval is likely due to the relatively heightened baseline activity during the training paradigm. Additionally, low frequency activation is noticeable in some channels before cue onset, which was likely due to anticipatory activity. The HG increase is followed by a low frequency rebound, which across many channels (especially in the center of the grid) reaches 100 Hz. The approximate central sulcus location is delineated by a thick black line (CS) and widens at the top such that channels 111, 119, and 127 are over it. The pre-central sulcus is delineated by a thick green line (Pre-CS).

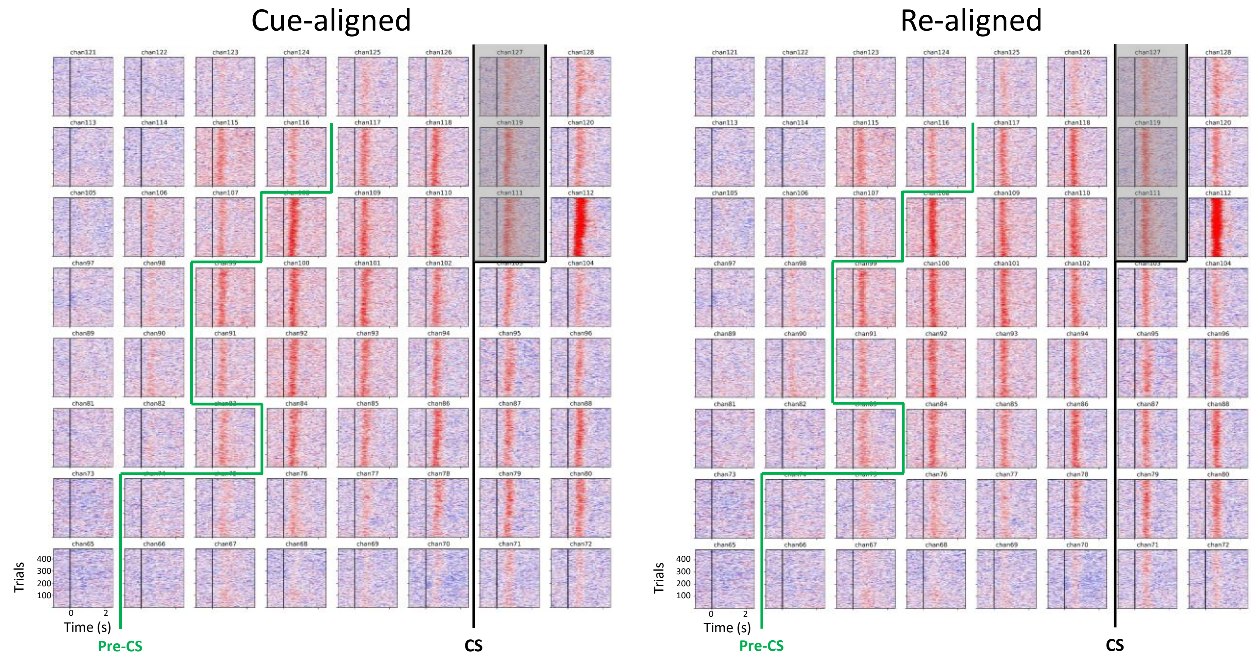

Supplementary Fig. 3 | Alignment of power trial rasters. Across all training blocks used for the original click detector, the cue-aligned HG power (110-170 Hz) for each trial is shown for all channels in the upper-limb grid (Cue-aligned). To account for inter-trial variability (especially for reaction delay in attempted movements), HG power between -1 s and 2.5 s post-cue from channels 86, 88, 94, 101, 102, 108, 109, 110, 112 were used to compute a per-trial shift, which was then used to re-align the HG power across all channels (Re-aligned). The approximate central sulcus location is delineated by a thick black line (CS) and widens at the top such that channels 111, 119, and 127 are over it. The pre-central sulcus is delineated by a thick green line (Pre-CS).

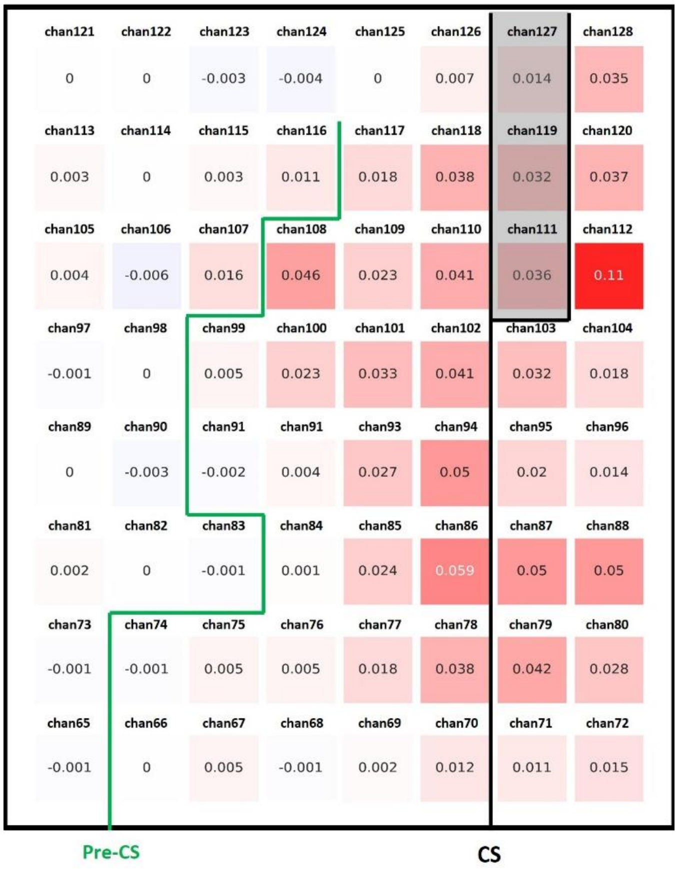

Supplementary Fig. 4 | Correlation increase of HG power in re-aligned signals. The change in correlation between the re-aligned and cue-aligned HG power trials for each electrode of the upper limb grid. Inter-trial HG power correlations generally increased for all electrodes. Electrodes in the speech grid (not shown) had negligible changes in inter-trial HG power correlation. The central sulcus (CS) is delineated by a thick black line and widens at the top such that channels 111, 119, and 127 are over it. The pre-central sulcus (Pre-CS) is delineated by a thick green line.

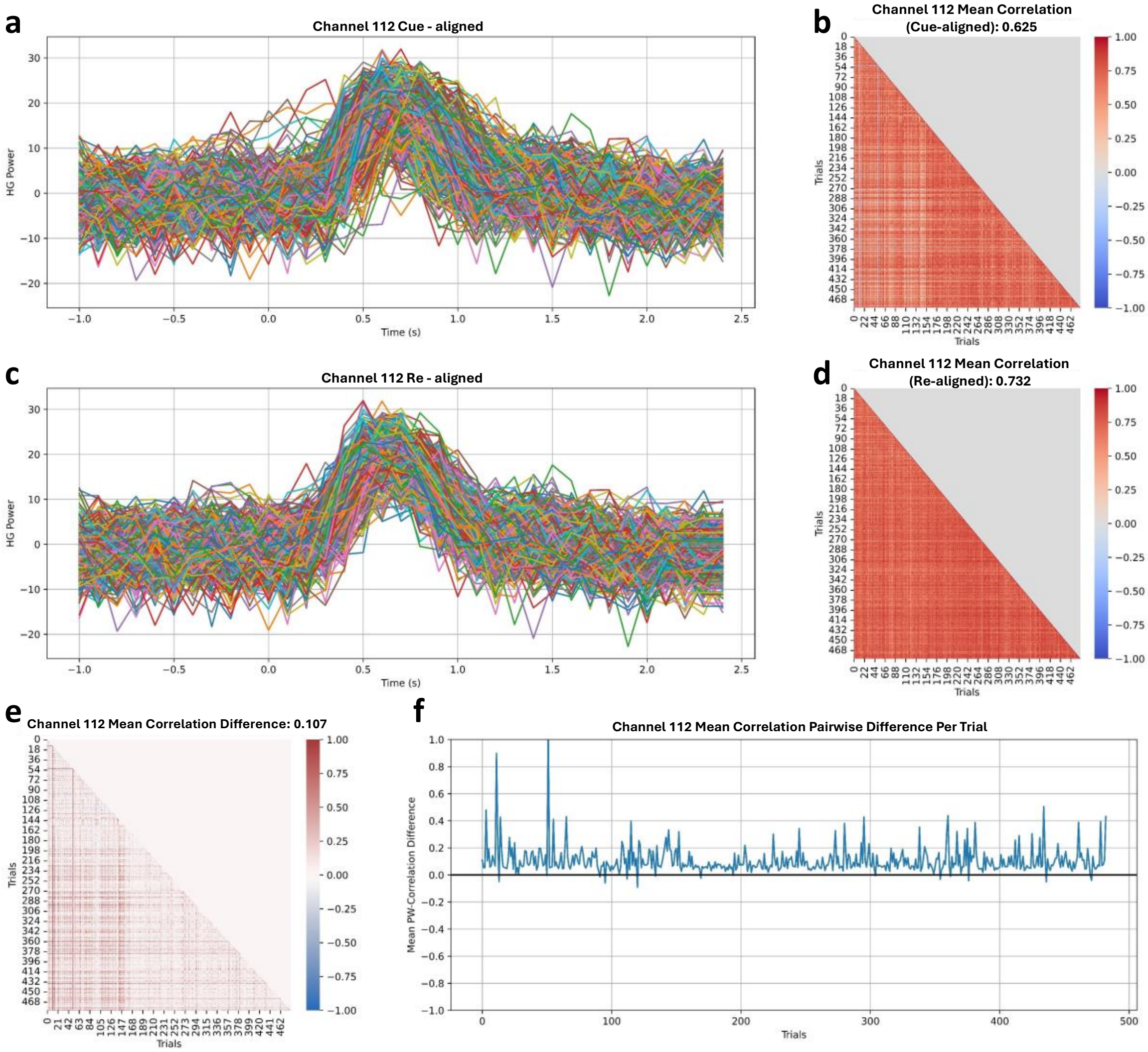

Supplementary Fig. 5 | Correlation analysis of channel 112 using re-aligned trials. Channel 112 is presented as an example of increased inter-trial correlation of HG power after re-alignment. (**a**) Cue-aligned HG power across all trials. (**b**) Pair-wise correlation between all trials for channel 112. The mean correlation is computed by taking the mean of all inter-trial correlation values and is computed similarly in (**d**) and (**e**). (**c**) Re-aligned HG power across all trials. (**d**) Increased pair-wise correlation between nearly all trials. (**e**) Difference between Re-aligned and Cue-aligned HG power across all trials. (**f**) Mean pair-wise correlation difference for each trial. For one trial, the mean pair-wise correlation change is computed by taking the mean of correlations between the HG power for that trial with those from all other trials.

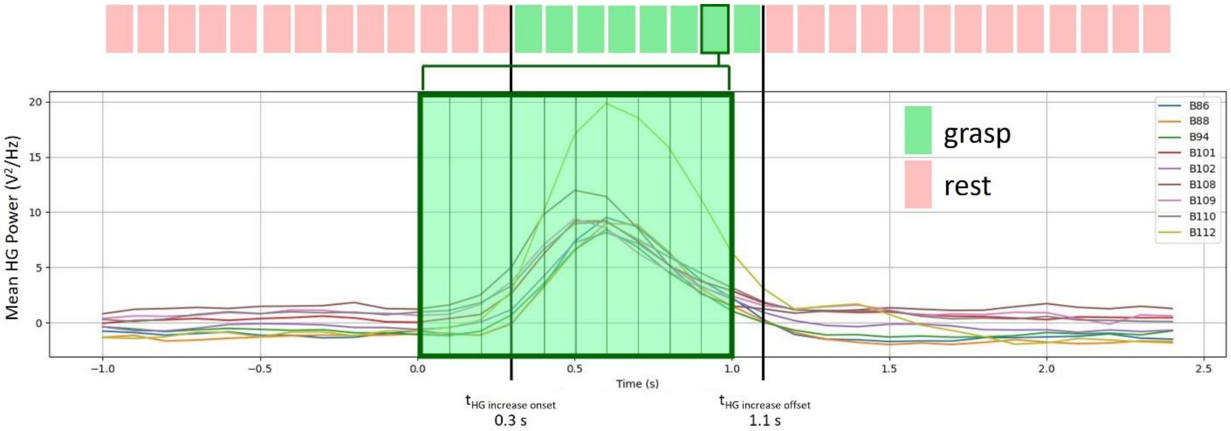

Supplementary Fig. 6 | Labeling trial-averaged re-aligned HG power. Trial-averaged re-aligned HG power is shown for each of the channels that were used to compute the per-trial shift. These averaged traces were used to determine the bounds for grasp labels on a per-trial basis. For each trial, each sample between 0.3 s and 1.1 s post-cue was labeled grasp while all other time points were labeled rest. For the training label at each time point, the corresponding training data is the 1 s sequence (previous 10 samples) of historical time features up until and including the time point of the that training label. For example, the training data for the grasp label at 1.0 s (dark green outline) is the 1 s sequence of historical time features from 0.1 to 1.0 s (large box with dark green outline). Though only the HG power traces for the above subset of channels were used to inform the assignment of grasp training labels, features from all channels were used as training data.

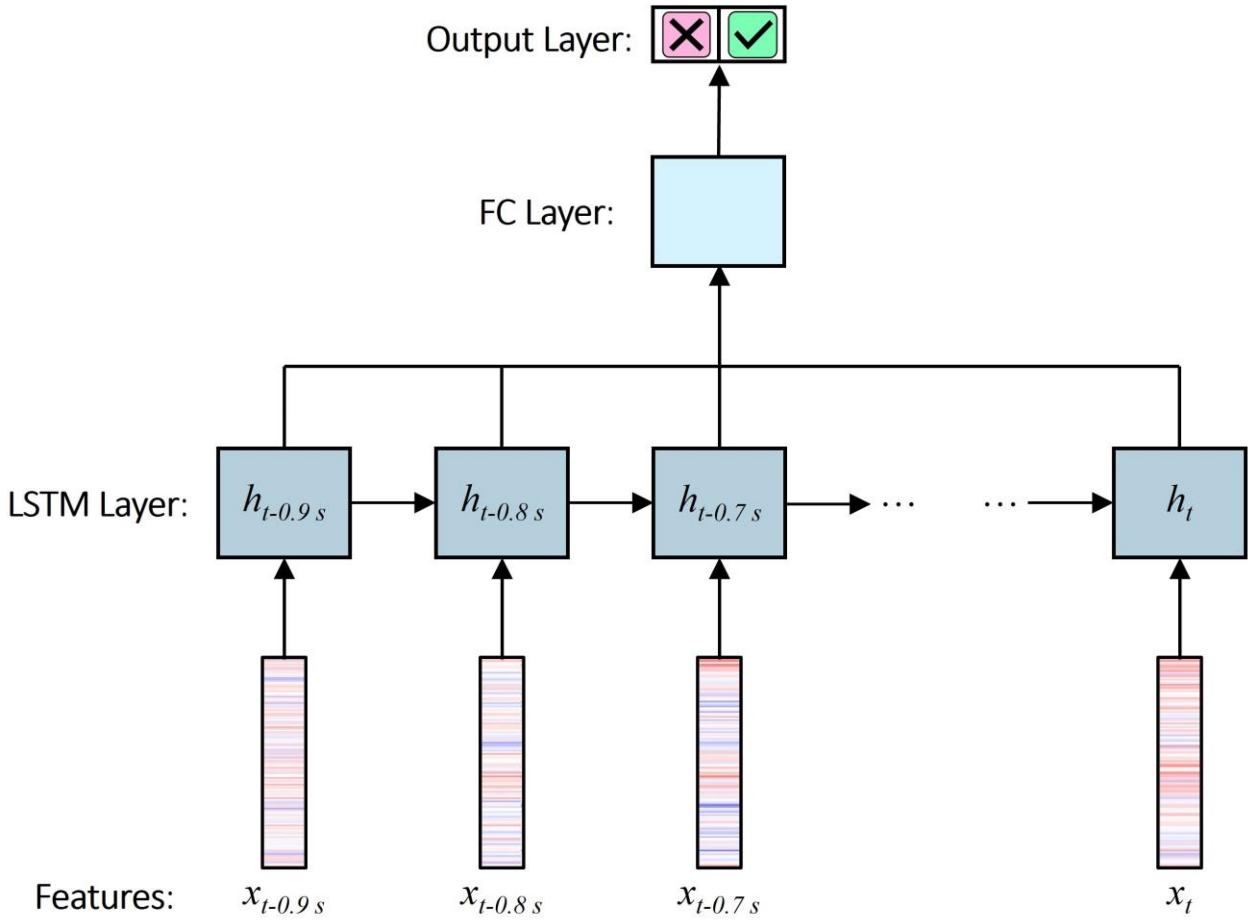

Supplementary Fig. 7 | Classification model architecture. We used a recurrent neural network that utilizes long short-term memory (LSTM) cells in a many-to-one configuration to predict output probabilities. The architecture of the network is comprised of 3 layers: 1 LSTM layer with 25 units followed by 2 fully-connected layers with 10 and 2 units, respectively, that incorporate eLU and softmax activation functions for non-linear transformations. We trained this network on high gamma power sequences with a fixed length of 1 s and a frame size of 100 ms using backpropagation through time (BPTT). Here, we relied on the Adam optimizer with an initial learning rate of 10e-4. In total, the network was trained for 75 epochs with a batch size of 45.

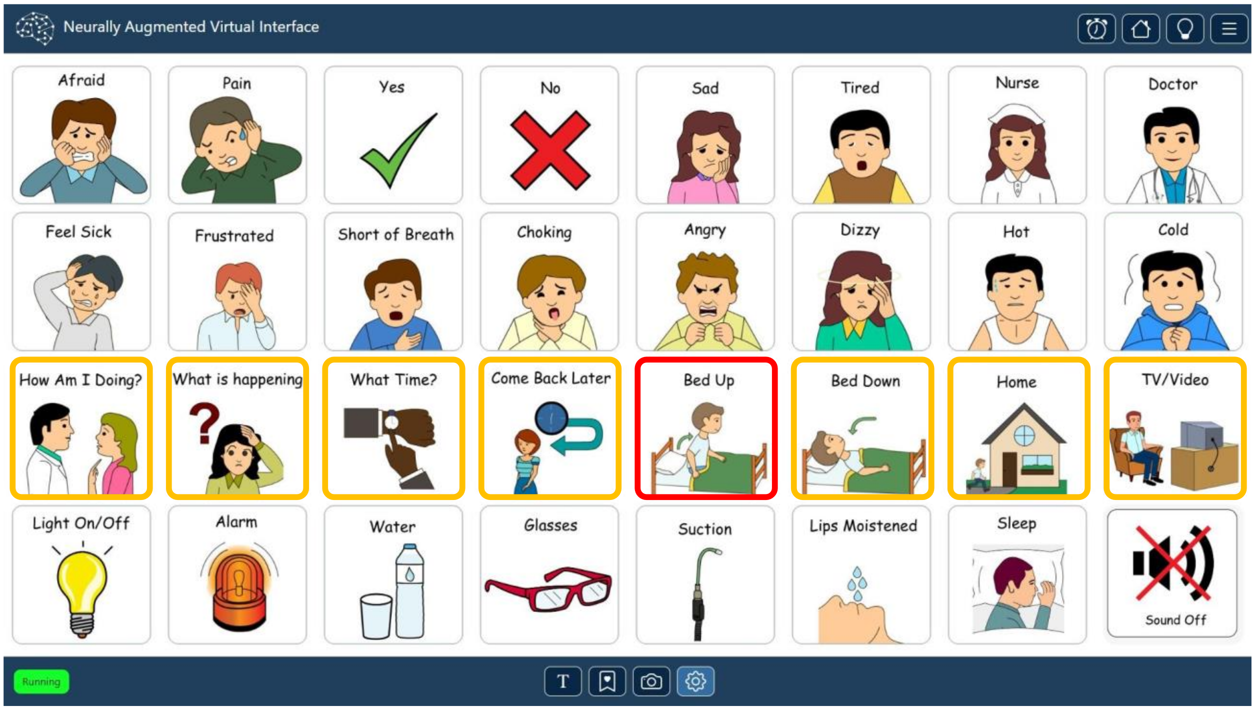

Supplementary Fig. 8 | User interface of communication board. The participant was instructed to select an experimenter-cued graphical button from a 4x8 grid by timing his clicks to the appropriate highlighted row or column during the switch scanning cycle. Switch scanning started by sequentially highlighting each row in green (not shown) for 1.5 s until the participant clicked on the row containing the cued button. Once a row was selected, all eight buttons in that row became outlined in yellow and then each column was sequentially highlighted for 1 s in red. Once the participant selected a button by clicking, the button became green for 1 s before the switch scanning process was reset at the first row and the participant received another cue. Pictograms were obtained from https://communicationboard.io/[8] and ARASAAC (https://arasaac.org/)[9].

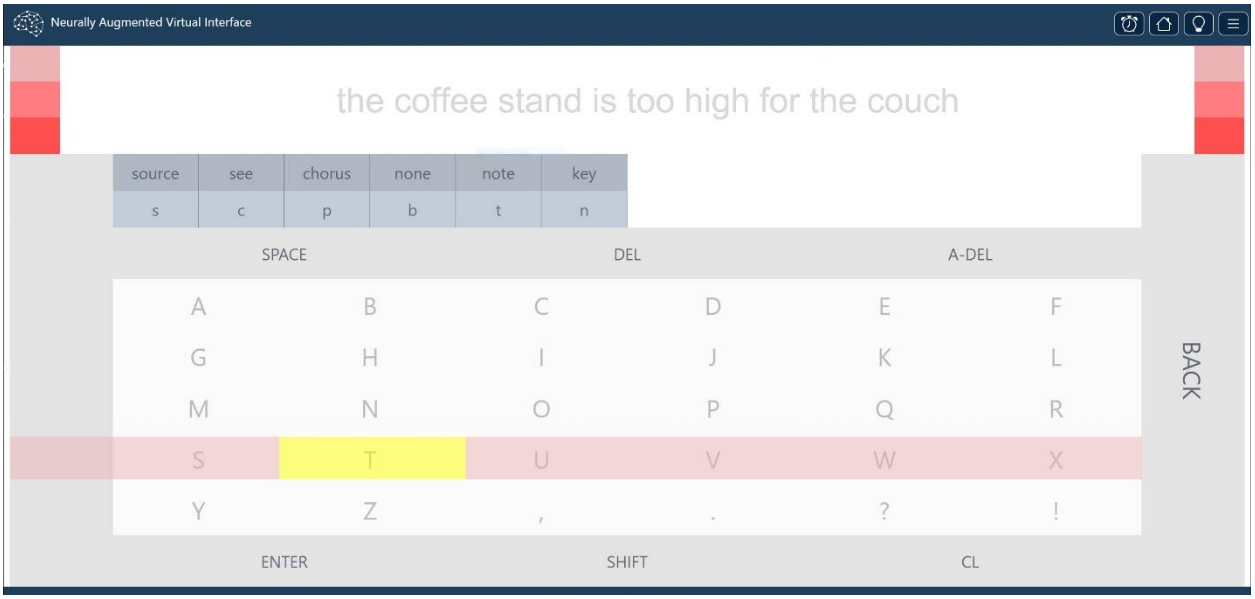

Supplementary Fig. 9 | User interface of switch scanning speller application. The participant was instructed to spell the sentence prompt (pale gray text) by timing his clicks to the appropriate highlighted row or column during the switch scanning cycle. The switch scanning process started by sequentially highlighting the three red pre-selection markers on either side of the sentence, where each highlight lasted 1 s. This was to allow the participant a brief preparation period in case he wanted to select row 1. Rows 1-8 were then scanned sequentially until the participant clicked on the row containing the appropriate letter or word. Rows 1 and 2 displayed the language model's most likely words and letters based on what the participant had already spelled. Row 3 allowed the participant to add a space, delete a letter or space, or delete an autocompleted word or letter (SPACE, DEL, and A-DEL keys, respectively). Rows 4-8 contained all alphabetical letters (and some grammatical symbols) in case the desired letter was not suggested in Row 2. Once a row was selected, the gray pre-selection column on the left was highlighted in yellow for 1 s to allow the participant a brief preparation period in case he wanted to select column 1. Buttons in the selected row were then sequentially highlighted in yellow for 1 s. At the end of each sequence of column highlights, the BACK key was highlighted to allow the participant to exit the row if it was selected accidentally. Clicking on any button (which would highlight it green for 1 s) within any row would reset the switch scanning process to Row 1. The participant finalized the sentence by selecting the ENTER button in Row 8. The SHIFT (for letter capitalization) and CL buttons (for erasing the spelled words) were functional but not used during the testing sessions. If the participant had not selected any of the eight rows, the switch scanning process was reset to Row 1.

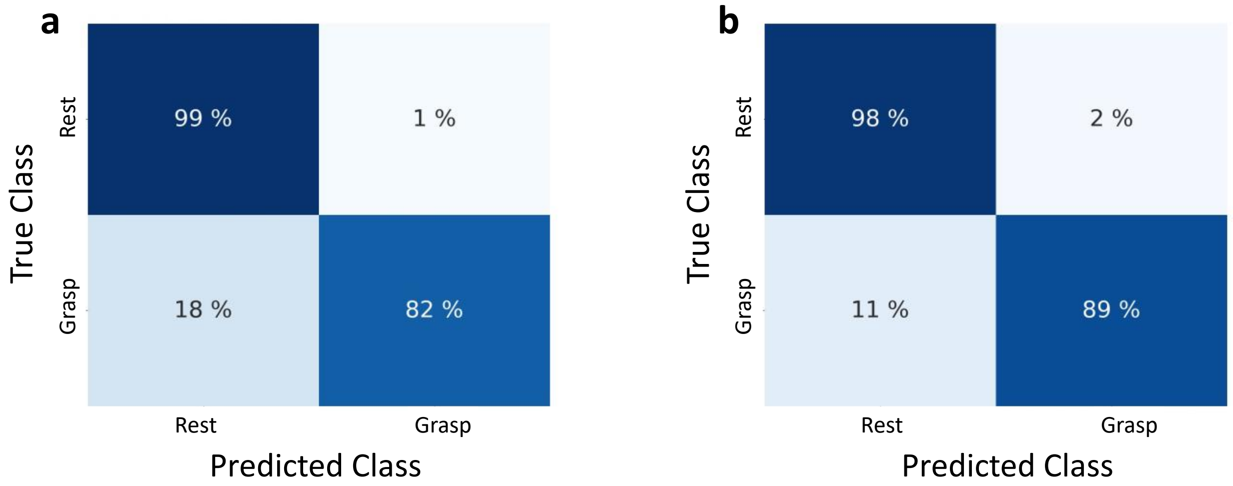

Supplementary Fig. 10 | Offline confusion matrices of spelling blocks. The confusion matrices on a sample-by-sample basis of all spelling blocks where the original fixed click detector operated with a 7-vote (**a**) and 4-vote (**b**) threshold.

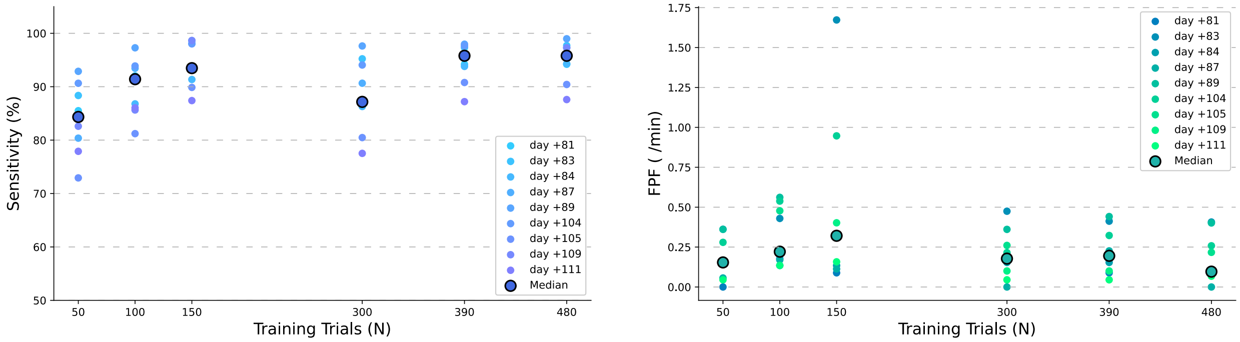

Supplementary Fig. 11 | Click detector performance as a function of training data. Simulated sensitivity (**a**) and false positive frequency (FPF) (**b**) are shown in relation to the number of trials used for training the corresponding click detector. For a specific number of training trials, the simulated sensitivity and FPF of the click detector are shown across all sessions in which the click detector operated with a 4-vote threshold (pale markers). The simulated median sensitivity and FPF values are shown as bold markers.

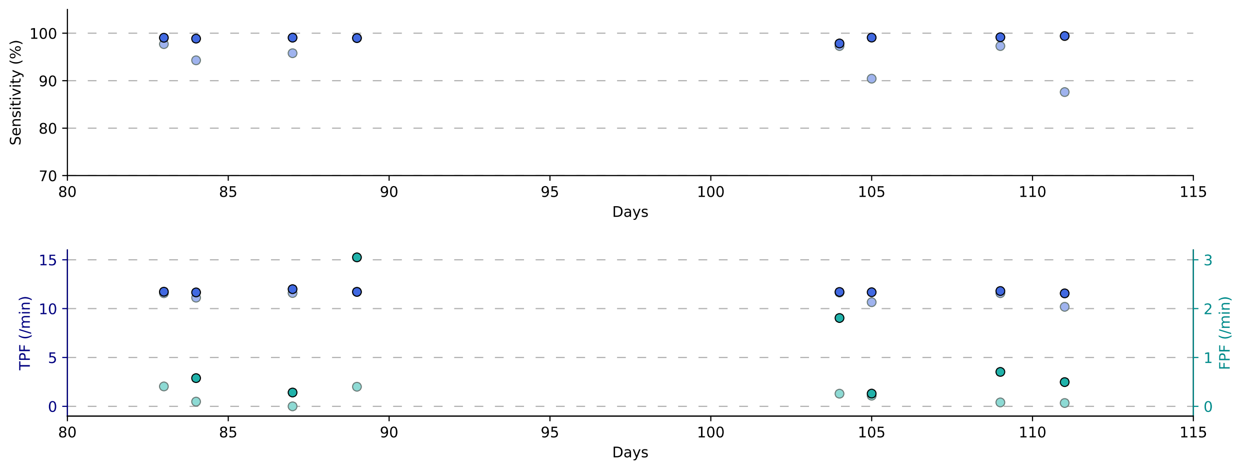

Supplementary Fig. 12 | Click detector performance using updated models. For all metrics, only spelling sessions in which the click detector operated with a 4-vote threshold are used. Dark and pale markers represent simulated metrics using the updated and original fixed click detectors, respectively. (**a**) Simulated sensitivity of click detection for each session. (**b**) Simulated true-positive and false positive frequencies (TPF and FPF) measured as detections per minute.

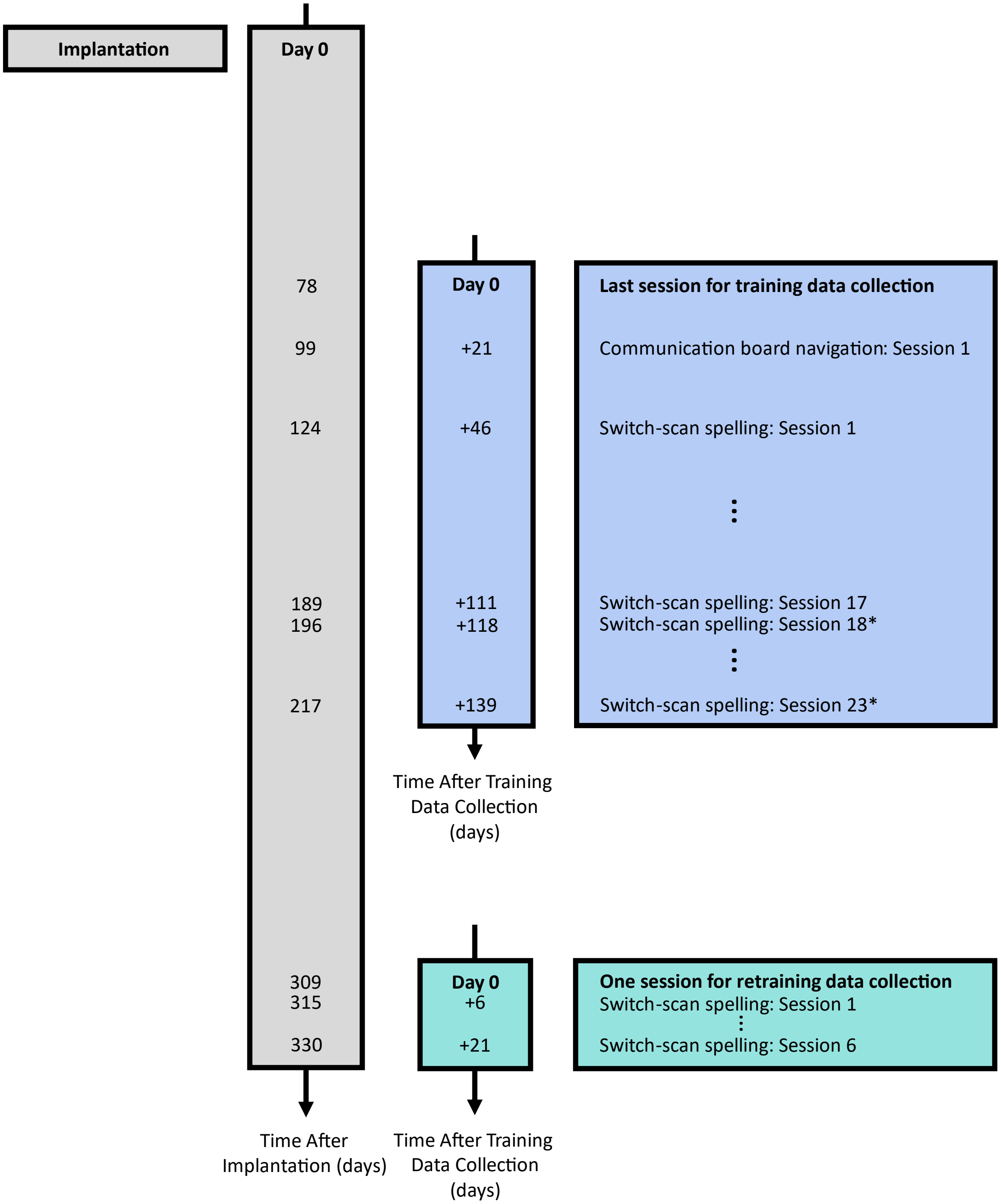

Supplementary Fig. 13 | Timelines of click use after training. Timeline of training and testing sessions relative to the surgical implantation (grey) where the implantation date is denoted as Day 0. Timeline of click usage with the communication board and spelling interface (blue) relative to the last session of data collection for training the original click detector. Timeline of click usage with the spelling interface (teal) relative to the only session of data collection for retraining a new click detector model. The last day of data collection relative to the original and retrained models is denoted by Day 0. Between Days 217 and 309 post-surgical implantation we investigated possible causes for the signal deviation. Note asterisks (Sessions 18-23 in which the original click detector was used) represent sessions in which click detection performance was suboptimal.

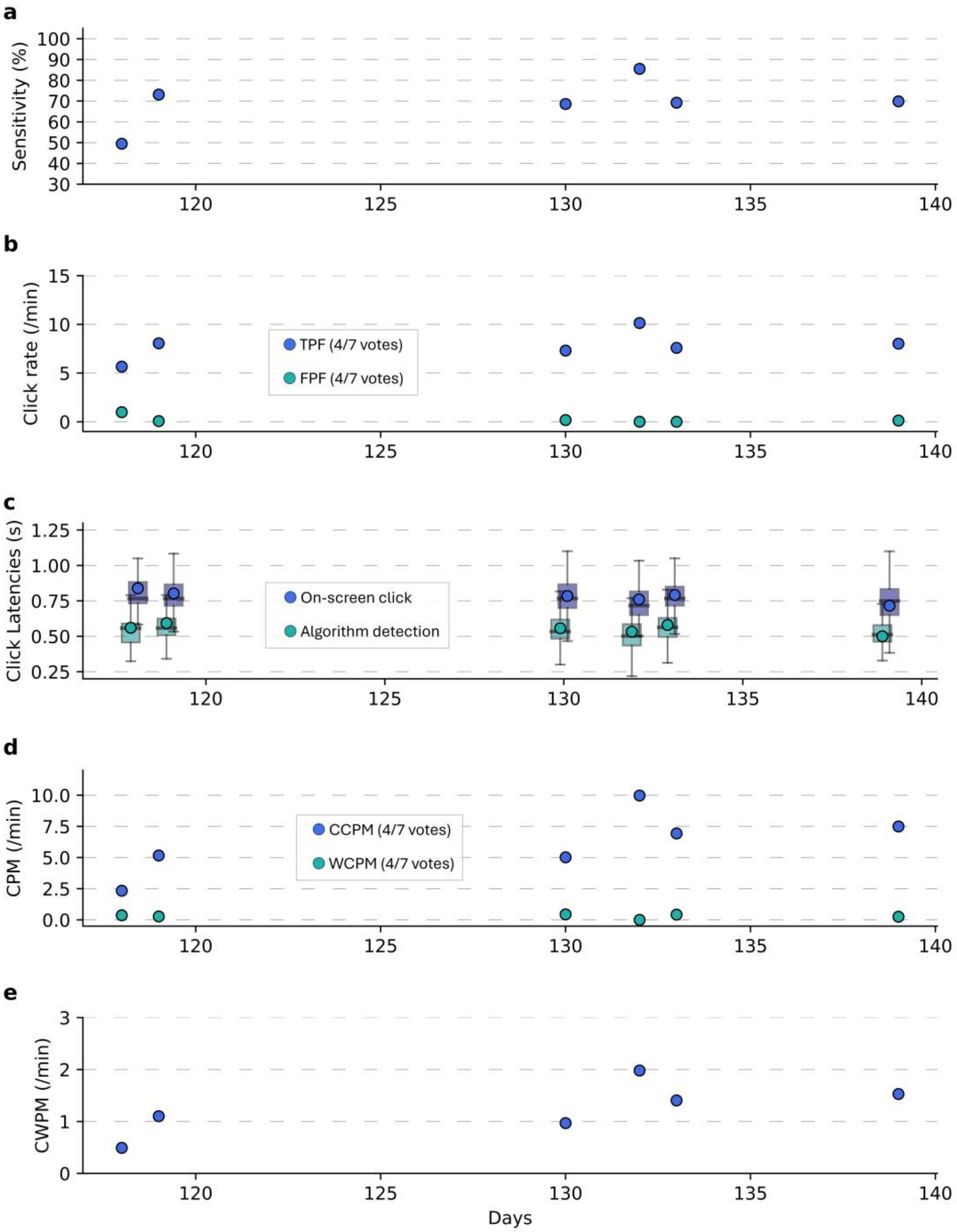

Supplementary Fig. 14 | Performance decline roughly 4 months post-training. For each day a 4-vote threshold was used. (**a**) Sensitivity of grasp detection for each session. (**b**) True positive and false-positive frequencies (TPF and FPF, which are represented by blue and green markers, respectively) measured as detections per minute. (**c**) Latencies of grasp onset to correct algorithm detection (green markers) and on-screen click (blue markers). For each session, there were 46, 117, 83, 307, 162, and 320 correctly detected clicks, respectively. Mean latencies are shown as circular markers that are overlayed on top of box-and-whisker plots. The distribution of latencies across all spelling blocks in a session was used to compute the mean latency and box-and-whisker plot for that session. For each box-and-whisker plot, the median is shown as the center line, the quartiles are shown as the top and bottom edges of the box, and the whiskers are shown at 1.5 times the interquartile range. (**d**) Characters per minute (CPM) are assessed in terms of correct and wrong characters per minute (CCPM and WCPM, which are represented by blue and green markers, respectively). (**e**) Correct words per minute (CWPM).

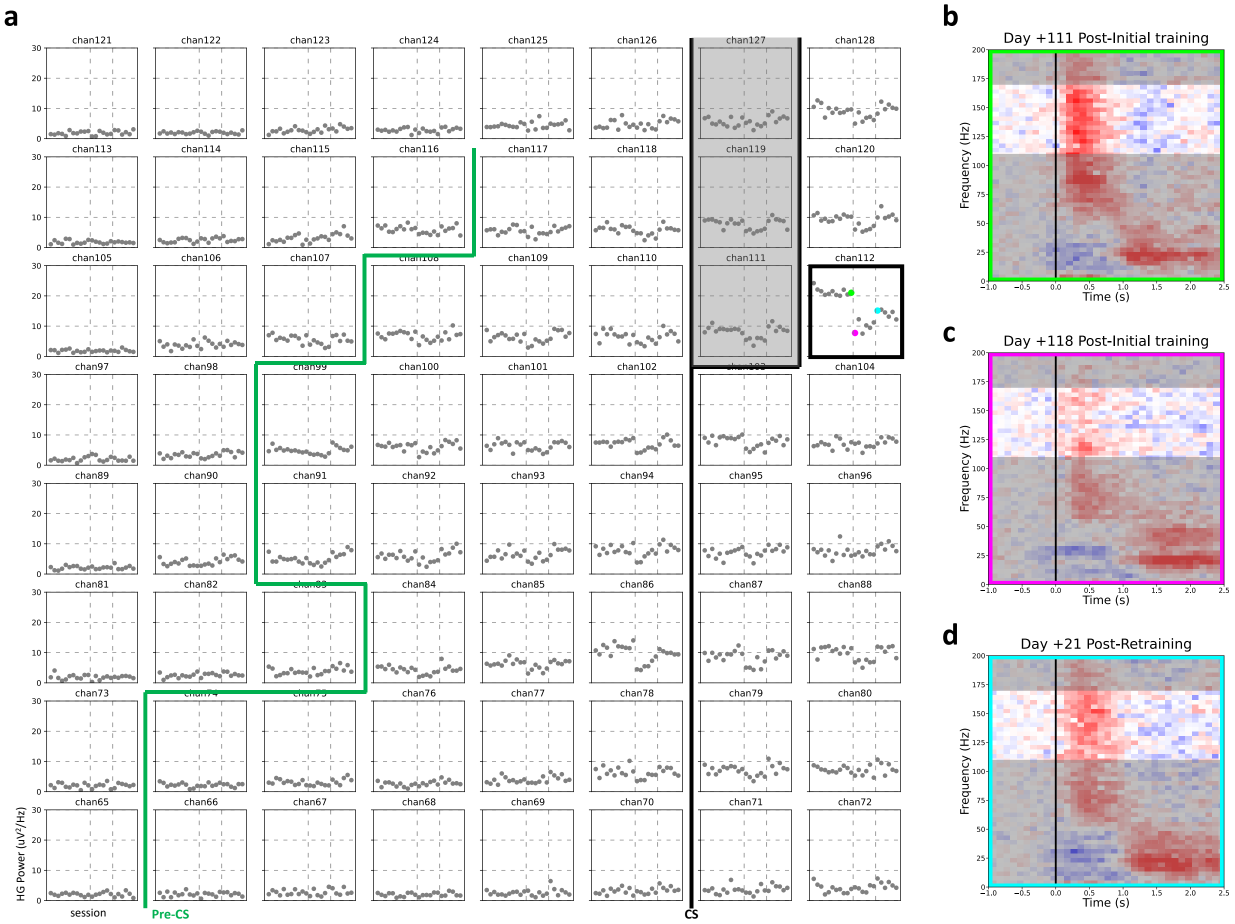

Supplementary Fig. 15 | Decrease in movement-aligned high gamma (HG) event related synchronization (ERS). (**a**) For each channel, one dot represents the maximum value of the median HG ERS from the first 30 movement-aligned trials. The horizontal axis represents spelling sessions. The two dashed vertical lines for each channel split these values into three regions: left) values prior to performance drop while using the original fixed click detector; middle) values during the performance drop while using the original fixed click detector; right) values after retraining a new fixed click detector. The largest decrease in HG ERS was observed in channel 112 (outlined in black), the most salient channel of the original click detection model. The green, pink, and blue dots are the maximum values of the HG ERS from the last session prior to performance drop, first session during the observed decreased performance, and first session using the newly trained click detector, respectively. The central sulcus is delineated by a thick black line (CS) and widens at the top such that channels 111, 119, and 127 are over it. The pre-central sulcus is delineated by a thick green line (Pre-CS). (**b**)-(**d**) show the trial-averaged spectral power changes in channel 112, with the HG frequency band (110-170 Hz) highlighted. Trial-averaged spectral power changes from the first 30 movement-aligned trials of the last spelling session prior to performance drop (**b**), the first spelling session with the observed decreased performance (**c**), and the first spelling session after training a new click detector (**d**).

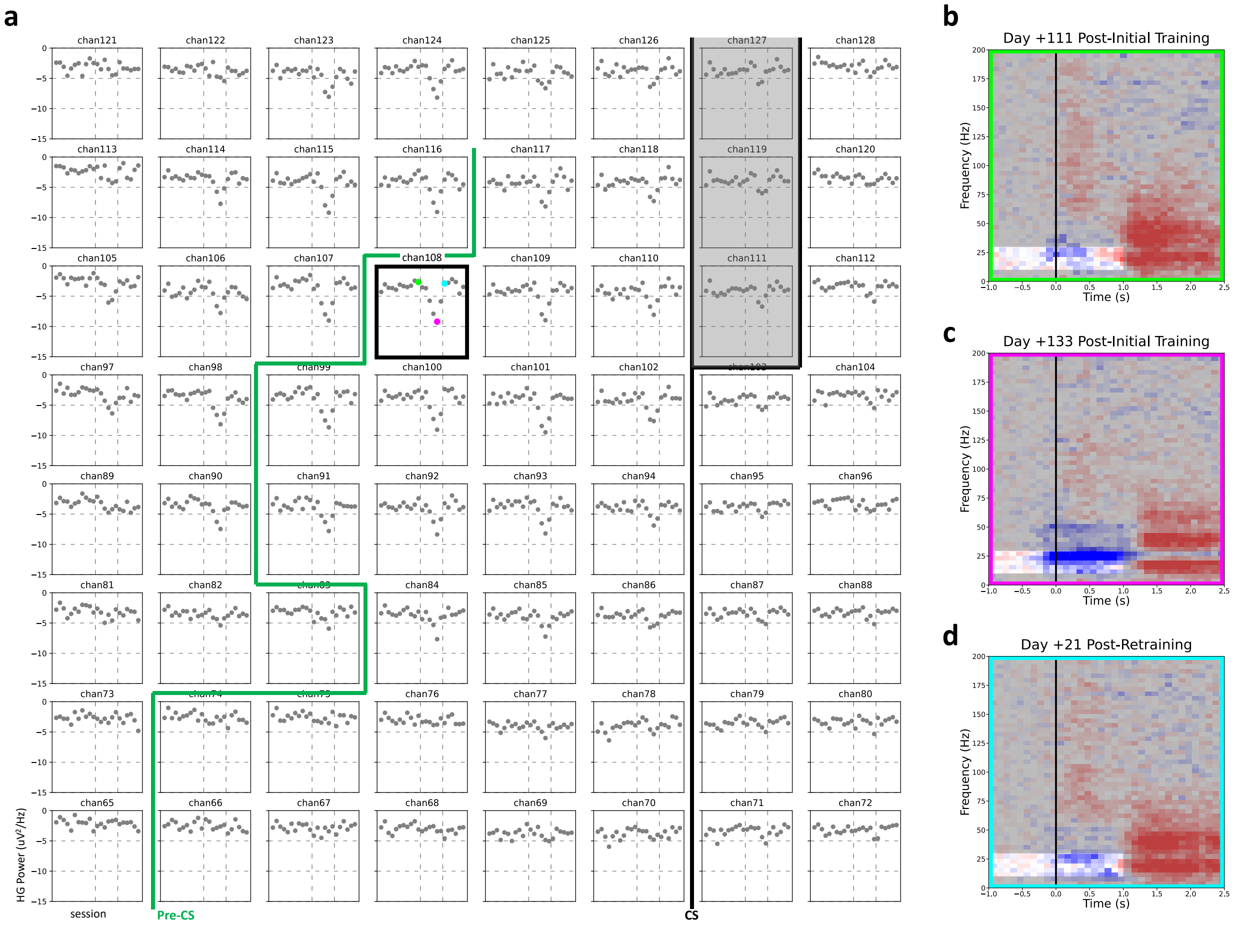

Supplementary Fig. 16 | Increase in low frequency (10-30 Hz) event-related desynchronization (ERD). (**a**) For each channel, one dot represents the nadir of the median low frequency ERD from the first 30 movement-aligned trials. The horizontal axis represents spelling sessions. The two dashed vertical lines for each channel split these values into three regions: left) values prior to performance drop while using the original fixed click detector; middle) values during the performance drop while using the original fixed click detector; right) values after retraining a new fixed click detector. The largest low frequency ERD was observed in channel 108 (outlined in black), the second-most salient channel of the original click detection model. The green, pink, and blue dots are the minimum values of low frequency ERD from the last session prior to performance drop, the fifth session with the observed decreased performance, and the first session using the newly trained click detector, respectively. The central sulcus is delineated by a thick black line (CS) and widens at the top such that channels 111, 119, and 127 are over it. The pre-central sulcus is delineated by a thick green line (Pre-CS). (**b**)-(**d**) show the trial-averaged spectral power changes from channel 108, with the low frequency band (10-30 Hz) highlighted only in the time range of ERD. Trial-averaged spectral power changes of the first 30 movement-aligned trials from the last spelling session prior to performance drop (**b**), the fifth spelling session with the observed decreased performance (**c**), and the first spelling session after training a new click detector (**d**).

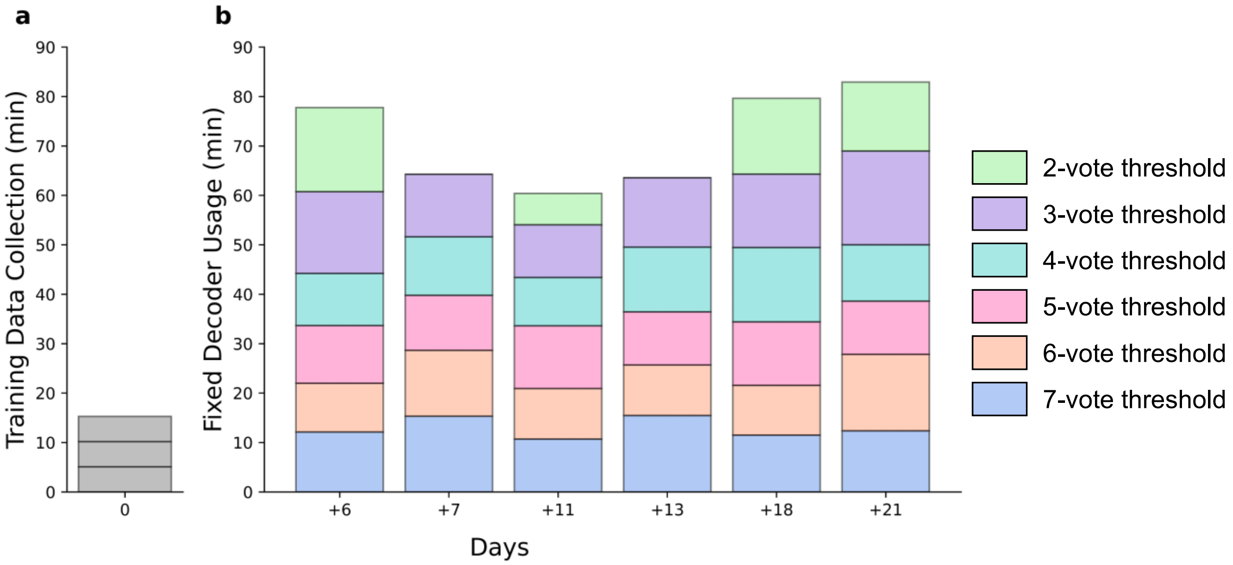

Supplementary Fig. 17 | Long-term use of a fixed retrained click detector. (**a**) Training data was collected on one session where the three sub-bars each represent a separate block of training data collection. (**b**) The retrained fixed click detector was used by the participant with a nearly complete range of voting thresholds on almost all days after retraining (a 1-vote threshold made the click detector unusable due to the high frequency of false positive detections). For each day, each sub-bar represents a separate spelling block of 3 sentences. The horizontal axis spanning both (**a**) and (**b**) represents the number of days after the last day of training data collection (Day 0).

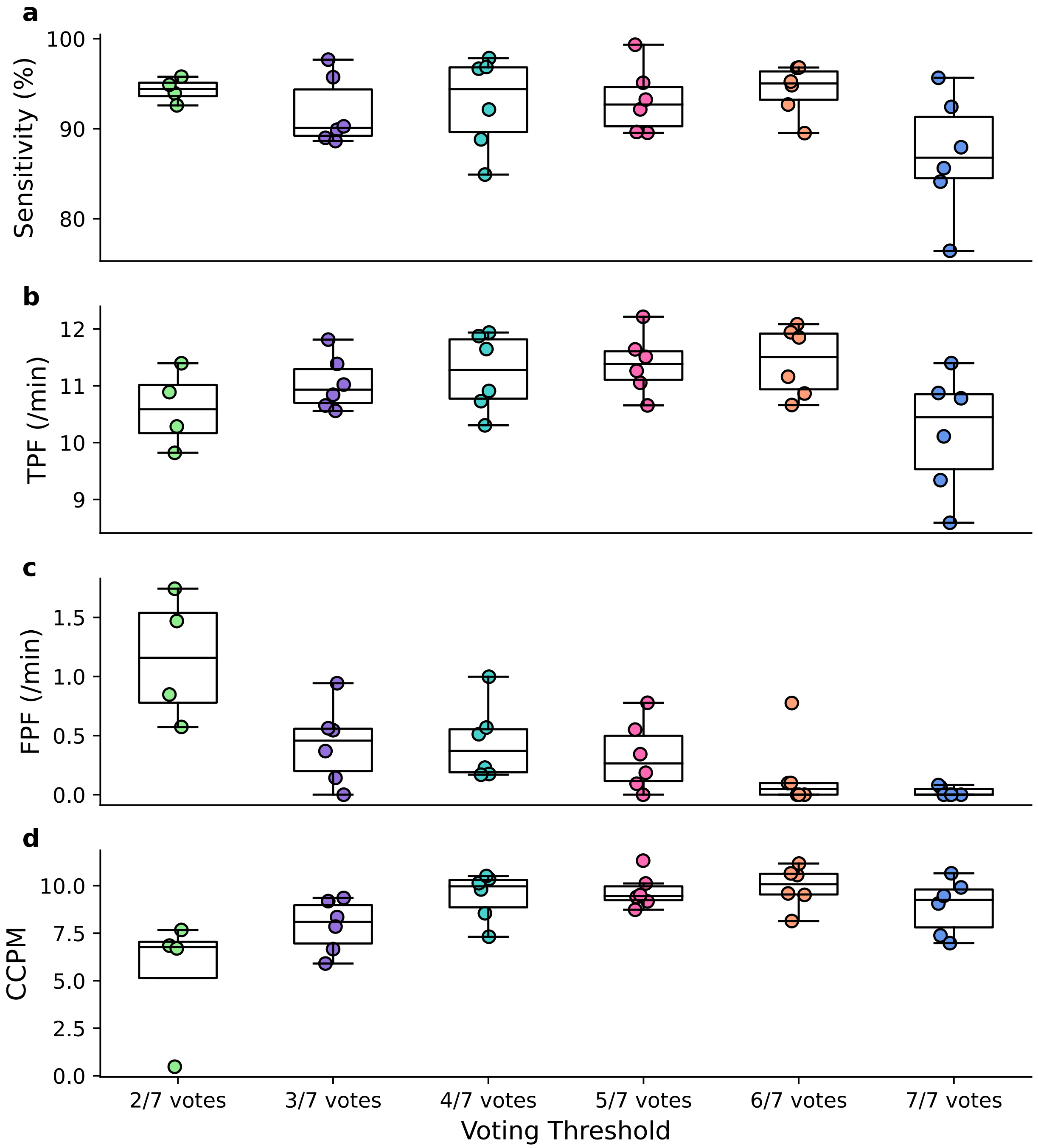

Supplementary Fig. 18 | Performance evaluation across all voting thresholds. Voting thresholds ranging from 3 to 7 votes were evaluated on 6 sessions whereas the 2-vote threshold was evaluated only during 4 sessions. For each boxplot, the median is shown as the center line, the quartiles are shown as the top and bottom edges of the box, and the whiskers are shown at 1.5 times the interquartile range. Sensitivity of grasp detection (**a**), true positive frequency (**b**), false positive frequency (**c**), and CCPM (**d**) observed with each voting threshold. Note that all above evaluations were performed using the retrained fixed click detector.

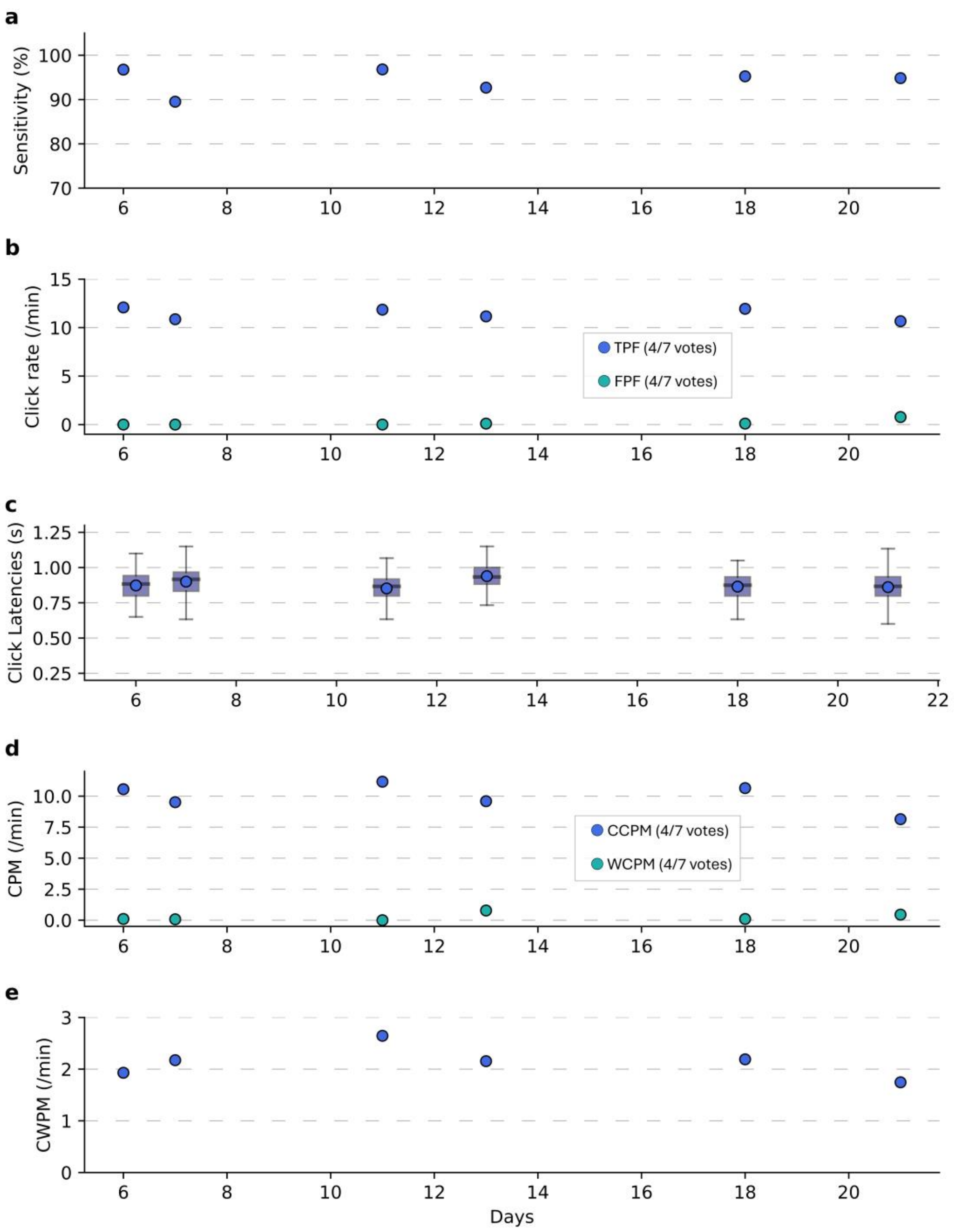

Supplementary Fig. 19 | Switch scan spelling performance with a retrained fixed click detector. All performance metrics are shown using the 6-vote threshold. (**a**) Sensitivity of grasp detection for each session. (**b**) True positive and false positive rates (TPF and FPF, which are represented by blue and green markers, respectively) measured as detections per minute. (**c**) Latencies of grasp onset to correct on-screen click (blue markers). For each session, there were 119, 145, 121, 114, 120, and 165 correctly detected clicks, respectively. Mean latencies are shown as circular markers that are overlayed on top of box-and-whisker plots. The distribution of latencies across all spelling blocks in a session were used to compute the mean latency and box-and-whisker plot for that session. For each box-and-whisker plot, the median is shown as the center line, the quartiles are shown as the top and bottom edges of the box, and the whiskers are shown at 1.5 times the interquartile range. (**d**) Characters per minute (CPM) are assessed in terms of correct and wrong characters per minute (CCPM and WCPM, which are represented by blue and green markers, respectively). (**e**) Correct words per minute (CWPM).

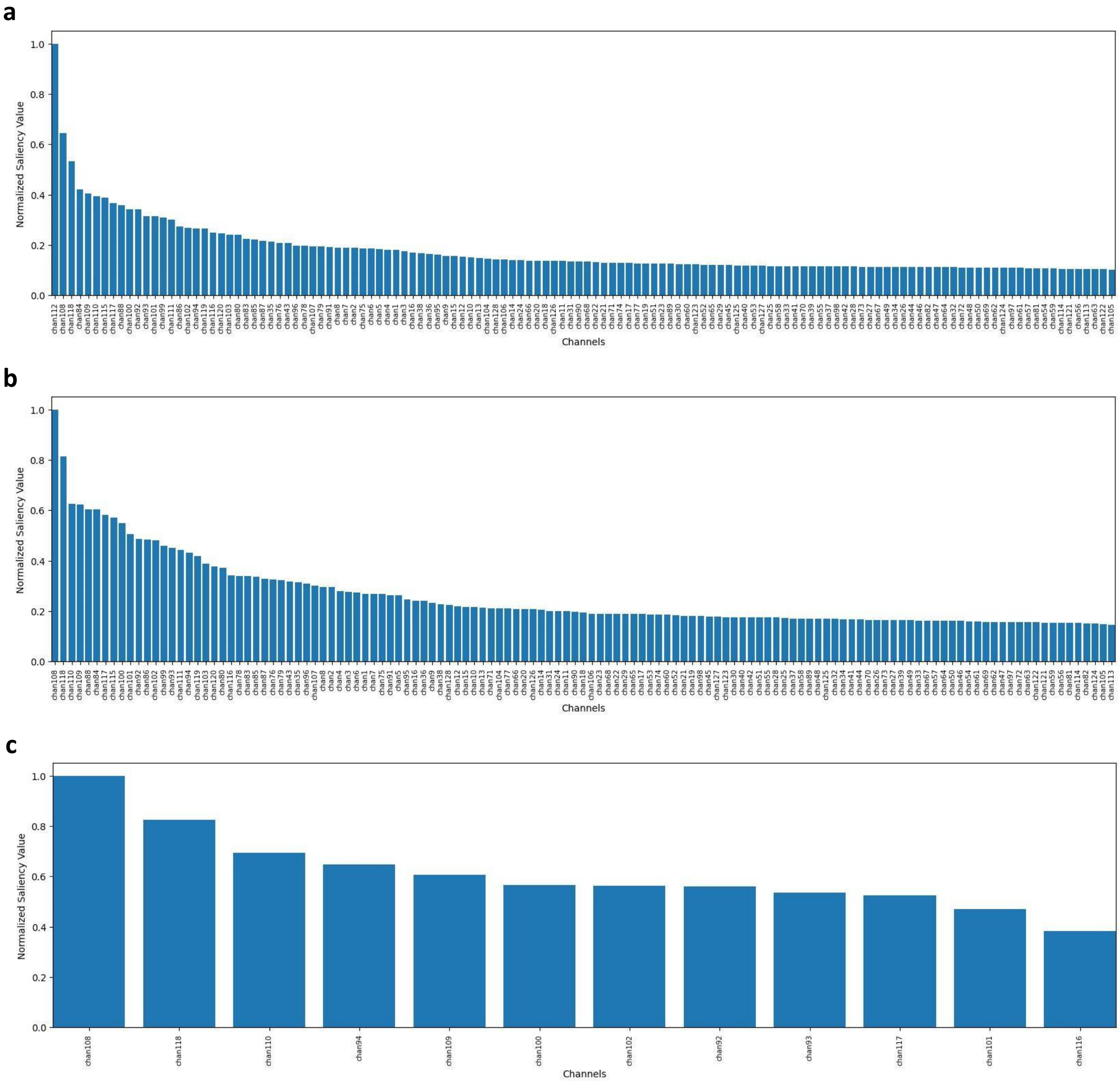

Supplementary Fig. 20 | Normalized saliency values. Normalized saliency values of each channel in descending order from a model trained on (**a**) all channels, (**b**) all channels excluding channel 112, and (**c**) a 4x3 subset covering cortical hand-knob.

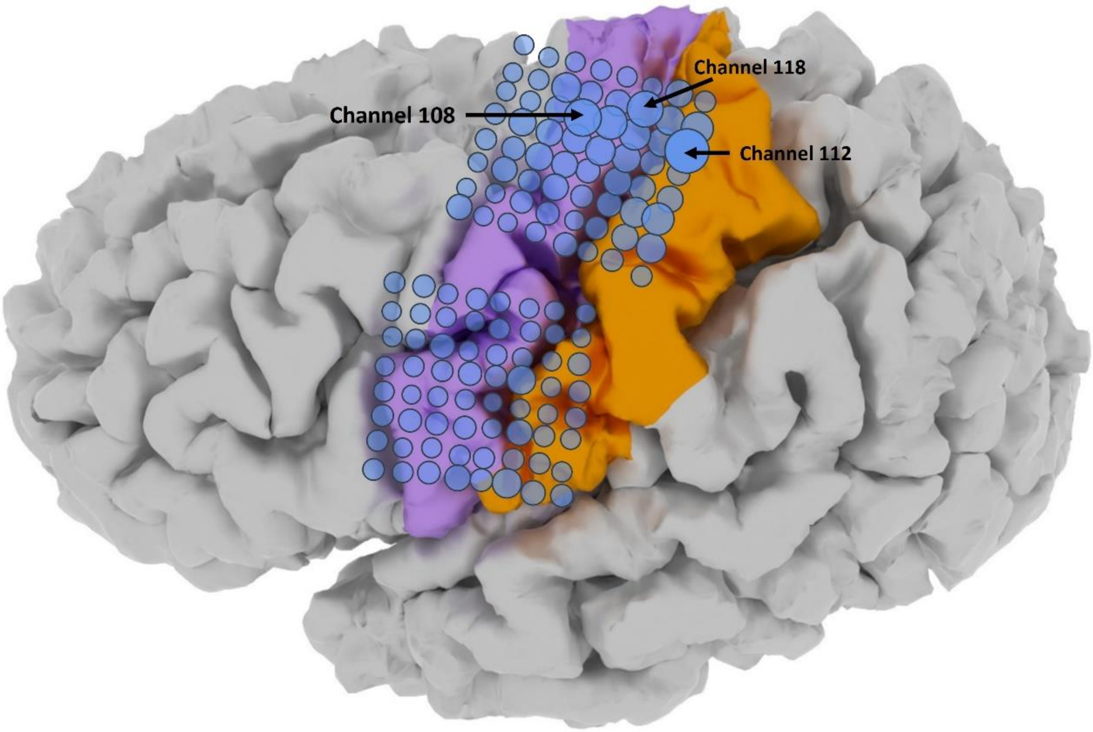

Supplementary Fig. 21 | Normalized saliency values during period with performance drop. Saliency maps for assessing which channels produced the most important HG features for attempted grasping during the period with the performance drop. Channels overlayed with larger and more opaque circles represent greater importance of that channel's HG features during attempted grasp. The channels with the three highest saliency values are marked.

Supplementary Tables

	Blocks	Trials/set	Number of sets	Post-set "rest"	ISI (seconds)	Total training time (min)
Session 1	1	50	1	No	3.5 - 4.5	3.77
Session 2	2	50	1	No	3.5 - 4.5	7.57
Session 3	1	50	3	Yes	3 - 6	14.09
Session 4	2	30	3	Yes	3 - 6	18.49

Supplementary Table 1 | Experimental parameters for training data collection. For each block during sessions 1 and 2 the participant performed one set of 50 trials whose ISIs were jittered between 3.5 - 4.5 s. For each block during sessions 3 and 4, we introduced a 90 s rest period after each of three sets of trials during which the participant was asked to remain still and fixate his eyes on a Rest stimulus. On session 4, we reduced the number of trials per set from 50 to 30 due to the participant's difficulty in focusing throughout the duration of the task. The ISI for sessions 3 and 4 was jittered between 3 - 6 s to further reduce anticipatory behavior. The above sessions were used to train the original click detector.

References

1. Metzger, S. L. *et al.* Generalizable spelling using a speech neuroprosthesis in an individual with severe limb and vocal paralysis. *Nat Commun* **13**, 6510 (2022).

2. Silversmith, D. B. *et al.* Plug-and-play control of a brain–computer interface through neural map stabilization. *Nat Biotechnol* **39**, 326–335 (2021).

3. Benabid, A. L. *et al.* An exoskeleton controlled by an epidural wireless brain–machine interface in a tetraplegic patient: a proof-of-concept demonstration. *The Lancet Neurology* **18**, 1112–1122 (2019).

4. Ajiboye, A. B. *et al.* Restoration of reaching and grasping movements through brain-controlled muscle stimulation in a person with tetraplegia: a proof-of-concept demonstration. *The Lancet* **389**, 1821–1830 (2017).

5. Vansteensel, M. J. *et al.* Fully Implanted Brain–Computer Interface in a Locked-In Patient with ALS. *N Engl J Med* **375**, 2060–2066 (2016).

6. Collinger, J. L. *et al.* High-performance neuroprosthetic control by an individual with tetraplegia. *The Lancet* **381**, 557–564 (2013).

7. Sundararajan, M., Taly, A. & Yan, Q. Axiomatic Attribution for Deep Networks. *Proceedings of the 34th International Conference on Machine Learning, PMLR* **70**, 3319–3328.

8. Ebin, J. & Delora, A. Communication Board. https://communicationboard.io.

9. Aragonese Center of Augmentative and Alternative Communication - ARASAAC. (2008). Recovered on 11th September 2024, from ARASAAC website: https://arasaac.org/.
