# Supplementary material for: A click-based electrocorticographic brain-computer interface enables long-term high-performance switch scan spelling
Source: Commun Med (Lond). 2024 Oct 22;4:207. doi: 10.1038/s43856-024-00635-3 (PMC11494178; doi:10.1038/s43856-024-00635-3)
Supplement: Supplementary file 3 — Description of Additional Supplementary Files [file 43856_2024_635_MOESM3_ESM.pdf]

Description of Additional Supplementary Files

File name: Supplementary Movie 1

File description: Switch scan control of a communication board. Using a switch scanning paradigm, the participant navigates to one of 32 symbols on the communication board. The participant must time his attempted grasps such that the click occurs when the desired row or column is highlighted in red. The scan rate across rows and columns is 1 switch per 1.5 s. Once a row or column is selected, it turns yellow or green, respectively.

File name: Supplementary Movie 2

File description: Switch scan spelling. Using a switch scanning paradigm, the participant navigates to the appropriate letter on the static keyboard or suggested letter or word to complete the prompted sentence (pale gray). The participant must time his attempted grasps such that the click occurs when the desired row or column is highlighted. The scan rate across rows and columns is 1 switch per 1 s. After the sentence is complete, the participant would click ENTER at the bottom of the screen.
