# Supplementary material for: A click-based electrocorticographic brain-computer interface enables long-term high-performance switch scan spelling
Source: Commun Med (Lond). 2024 Oct 22;4:207. doi: 10.1038/s43856-024-00635-3 (PMC11494178; doi:10.1038/s43856-024-00635-3)
Supplement: Supplementary file 6 — Reporting Summary [file 43856_2024_635_MOESM6_ESM.pdf]

nature portfolio

Corresponding author(s): Nathan Crone

Last updated by author(s): April 24, 2024

Reporting Summary

Nature Portfolio wishes to improve the reproducibility of the work that we publish. This form provides structure for consistency and transparency in reporting. For further information on Nature Portfolio policies, see our Editorial Policies and the Editorial Policy Checklist.

Statistics

For all statistical analyses, confirm that the following items are present in the figure legend, table legend, main text, or Methods section.

n/a	Confirmed	
☐	☒	The exact sample size (*n*) for each experimental group/condition, given as a discrete number and unit of measurement
☒	☐	A statement on whether measurements were taken from distinct samples or whether the same sample was measured repeatedly
☐	☒	The statistical test(s) used AND whether they are one- or two-sided<br>*Only common tests should be described solely by name; describe more complex techniques in the Methods section.*
☒	☐	A description of all covariates tested
☐	☒	A description of any assumptions or corrections, such as tests of normality and adjustment for multiple comparisons
☐	☒	A full description of the statistical parameters including central tendency (e.g. means) or other basic estimates (e.g. regression coefficient) AND variation (e.g. standard deviation) or associated estimates of uncertainty (e.g. confidence intervals)
☐	☒	For null hypothesis testing, the test statistic (e.g. *F*, *t*, *r*) with confidence intervals, effect sizes, degrees of freedom and *P* value noted<br>*Give P values as exact values whenever suitable.*
☒	☐	For Bayesian analysis, information on the choice of priors and Markov chain Monte Carlo settings
☒	☐	For hierarchical and complex designs, identification of the appropriate level for tests and full reporting of outcomes
☒	☐	Estimates of effect sizes (e.g. Cohen's *d*, Pearson's *r*), indicating how they were calculated

Our web collection on statistics for biologists contains articles on many of the points above.

Software and code

Policy information about availability of computer code

Data collection	Neural data was collected by Central Suite (version 7.0.5, Blackrock Neurotech) and was stored by BCI2000 (version 3.0.5) for offline analysis.
Data analysis	Model training and offline analysis were done using Python (version 3.9.13). The recurrent neural network was built using Keras with a TensorFlow backend (version 2.8.0). Real time decoding was done in Python (version 3.10.12) using the ezmsg messaging architecture (version 3.0.0).

For manuscripts utilizing custom algorithms or software that are central to the research but not yet described in published literature, software must be made available to editors and reviewers. We strongly encourage code deposition in a community repository (e.g. GitHub). See the Nature Portfolio guidelines for submitting code & software for further information.

Data

Policy information about availability of data

All manuscripts must include a data availability statement. This statement should provide the following information, where applicable:

- Accession codes, unique identifiers, or web links for publicly available datasets
- A description of any restrictions on data availability
- For clinical datasets or third party data, please ensure that the statement adheres to our policy

Source data for rendering the figures in the main text and supplementary information is publicly available at DOI 10.17605/OSF.IO/46SKB. Beginning immediately after publication, neural data from the study participant and the study protocol will be available from the corresponding author upon reasonable request.

Human research participants

Policy information about studies involving human research participants and Sex and Gender in Research.

Reporting on sex and gender	The study design allowed for both male and female candidates to be recruited as participants in the clinical trial. Due to the nature of this N-of-1 study, no sex- or gender-based analyses were performed. The participant's sex was self-reported as male.
Population characteristics	Our study participant was 61 years old at the time of implant and had been diagnosed with amyotrophic lateral sclerosis (ALS) 8 years prior.
Recruitment	Information about the clinical trial was disseminated through a variety of channels, including ClinicalTrials.gov, Facebook ads, presentations at regional ALS support groups and the Northeast Amyotrophic Lateral Sclerosis Consortium (NEALS), and outreach to faculty and staff in the Johns Hopkins ALS and Stroke Centers. After expressing interest in the trial or being referred to the study team, participants were interviewed by the PI or a member of the study team to determine their eligibility and were fully informed of the potential benefits and risks of participation prior to giving informed consent. Consented participants underwent extensive screening procedures including fMRI, neuropsychological evalution, and medical consultations prior to implantation and were considered enrolled upon the decision to proceed with implantation of the study device. Given the small number of participants (1 to date, of a total of 3 planned), and the exploratory nature of this phase I study, we do not believe that our recruitment procedures are likely to have biased or otherwise impacted our results.
Ethics oversight	The study protocol was reviewed and approved by Johns Hopkins University Institutional Review Board and by the US Food and Drug Administration (FDA) under an investigational device exemption (IDE). Additionally, we regularly reported to a data and safety monitoring board (DSMB) who were independent of the study.

Note that full information on the approval of the study protocol must also be provided in the manuscript.

Field-specific reporting

Please select the one below that is the best fit for your research. If you are not sure, read the appropriate sections before making your selection.

☒ Life sciences ☐ Behavioural & social sciences ☐ Ecological, evolutionary & environmental sciences

For a reference copy of the document with all sections, see nature.com/documents/nr-reporting-summary-flat.pdf

Life sciences study design

All studies must disclose on these points even when the disclosure is negative.

Sample size	Not applicable for an this N-of-1 study.
Data exclusions	No data was excluded from this analysis.
Replication	Neural data collection and processing as well as decoder performance were reproducible across sessions as the participant was able to repeatedly demonstrate control of clicks using neural signals from attempted hand movements to spell sentences. However, as this study reports on the first participant in this trial so far, further work is necessary to test reproducibility of these results with other participants.
Randomization	Group allocation was not possible as our study involved only one participant.
Blinding	Blinding to group allocation was not possible as our study involved only one participant.

Reporting for specific materials, systems and methods

We require information from authors about some types of materials, experimental systems and methods used in many studies. Here, indicate whether each material, system or method listed is relevant to your study. If you are not sure if a list item applies to your research, read the appropriate section before selecting a response.

Materials & experimental systems

n/a	Involved in the study
☒	☐ Antibodies
☒	☐ Eukaryotic cell lines
☒	☐ Palaeontology and archaeology
☒	☐ Animals and other organisms
☐	☒ Clinical data
☒	☐ Dual use research of concern

Methods

n/a	Involved in the study
☒	☐ ChIP-seq
☒	☐ Flow cytometry
☒	☐ MRI-based neuroimaging

Clinical data

Policy information about clinical studies

All manuscripts should comply with the ICMJE guidelines for publication of clinical research and a completed CONSORT checklist must be included with all submissions.

Clinical trial registration: NCT03567213

Study protocol: The study protocol will be available from the corresponding author upon reasonable request beginning 6 months and ending 24 months following article publication.

Data collection: The participant in this study was consented on December 14, 2021. He completed all pre-screening procedures and was enrolled on July 5, 2022. Data collection started on July 29th, 2022, after a three-week recovery period from the surgical implantation of the study device on July 7, 2022. Data collection sessions occurred three times per week at the Crone lab space in The Johns Hopkins Hospital. Each session lasted approximately four hours. At the time of this writing, research testing sessions are ongoing.

Outcomes: Due to the exploratory nature of the study, the primary outcomes are stated in general terms evaluating the safety and recording viability of the implanted device, as well as preliminary assessment of BCI functionality enabled by the device. The metrics and statistics for BCI functionality are not predefined due to the limited number of trial participants and the exploratory nature of the study with respect to strategies for achieving BCI functionality. The secondary outcomes are the success rate and latency reported in terms of click detection accuracy and time from attempted movement onset to click.
